# Unraveling the mechanism of furfural tolerance in engineered *Pseudomonas putida* by genomics

**DOI:** 10.3389/fmicb.2022.1035263

**Published:** 2022-10-20

**Authors:** Lihua Zou, Xinzhu Jin, Yuanming Tao, Zhaojuan Zheng, Jia Ouyang

**Affiliations:** Jiangsu Co-Innovation Center of Efficient Processing and Utilization of Forest Resources, College of Chemical Engineering, Nanjing Forestry University, Nanjing, China

**Keywords:** adaptive laboratory evolution, furan aldehydes, lignocellulose, *Pseudomonas putida*, whole-genome resequencing

## Abstract

As a dehydration product of pentoses in hemicellulose sugar streams derived from lignocellulosic biomass, furfural is a prevalent inhibitor in the efficient microbial conversion process. To solve this obstacle, exploiting a biorefinery strain with remarkable furfural tolerance capability is essential. *Pseudomonas putida* KT2440 (*P. putida*) has served as a valuable bacterial chassis for biomass biorefinery. Here, a high-concentration furfural-tolerant *P. putida* strain was developed *via* adaptive laboratory evolution (ALE). The ALE resulted in a previously engineered *P. putida* strain with substantially increased furfural tolerance as compared to wild-type. Whole-genome sequencing of the adapted strains and reverse engineering validation of key targets revealed for the first time that several genes and their mutations, especially for PP_RS19785 and PP_RS18130 [encoding ATP-binding cassette (ABC) transporters] as well as PP_RS20740 (encoding a hypothetical protein), play pivotal roles in the furfural tolerance and conversion of this bacterium. Finally, strains overexpressing these three striking mutations grew well in highly toxic lignocellulosic hydrolysate, with cell biomass around 9-, 3.6-, and two-fold improvement over the control strain, respectively. To our knowledge, this study first unravels the furan aldehydes tolerance mechanism of industrial workhorse *P. putida*, which provides a new foundation for engineering strains to enhance furfural tolerance and further facilitate the valorization of lignocellulosic biomass.

## Introduction

Lignocellulosic biomass from agricultural and forest residues represents one of the most abundant and readily available carbon resources on earth for sustainable bioproduction of fuels and chemicals (Brethauer and Studer, 2015; Wang et al., 2019). Pretreatment is generally the mandatory step to depolymerize recalcitrant lignocellulosic biomass to obtain monomeric sugars for microbial fermentation (Wang et al., 2013, 2017b). Consequently, a variety of toxic side-products such as weak acids (formic, acetic, and levulinic acids), furan aldehydes (furfural and 5-hydroxymethylfurfural; 5-HMF), and phenolics were inevitably formed during pretreatment which seriously inhibits subsequent cell growth and fermentation (Jiang et al., 2021). Of such inhibitory compounds, the dehydration products of pentoses, furfural is widely regarded as a major toxic compound due to its abundance and strong cytotoxicity (Wierckx et al., 2011; Zheng et al., 2022). It could potentiate the toxicity of hydrolysates by acting synergistically with other inhibitory compounds, such as organic acids or phenolics (Wang et al., 2016; Singh et al., 2019).

The effects of furfural toxicity on microbial cells are thought to be multifaceted. It can destroy the integrity of the cell membrane, affecting the rate of cell replication and ATP generation rate (Zaldivar et al., 1999; Almeida et al., 2007). Moreover, it damages DNA, inhibiting RNA and protein synthesis (Singh et al., 2019). It was reported to inhibit the glycolytic and fermentative enzymes (Wang et al., 2018). Furfural has been shown to severely affect intracellular redox metabolism, induce the accumulation of reactive oxygen species, and cause damage to cell organelles (Jilani et al., 2020; Li et al., 2022). To reduce the toxicity level of furfural, different strategies for engineering microbial tolerance against furan aldehydes have been reported (Nieves et al., 2015). Furfural tolerance was conferred in *Escherichia coli* (*E. coli*) by deleting NADPH-dependent genes *yqhD* and *dkgA* (Miller et al., 2009b), overexpressing NADH-dependent *fucO* (Wang et al., 2011), and increasing expression of transhydrogenase gene *pntAB* (Miller et al., 2009a). Furthermore, overexpression of polyamine transporter genes or expression of the small multidrug resistance (SMR) pumps made *E. coli* cells more resistant to furfural (Gosset, 2017; Kurgan et al., 2019). The mutant of global transcription sigma factor *rpoD* was found to increase furfural tolerance of *Zymomonas mobilis* (*Z. mobilis*) (Tan et al., 2015). Additionally, the co-expression of transhydrogenase gene *udhA* and alcohol dehydrogenase gene ZMO1771 in *Z. mobilis* also improved the furfural resistance of this bacterium (Wang et al., 2017b). In *Saccharomyces cerevisiae* (*S. cerevisiae*), overexpression of oxidoreductase genes *ADH1, ADH6*, or *ARI1* successfully imparted tolerance against furfural (Wang et al., 2017b). Although progress has been made by targeting various genetic traits for decades, the effect of these beneficial genetic traits is limited, and mechanisms for conferring tolerance remain to be investigated (Wang et al., 2013; Glebes et al., 2015). Further improving the tolerance of platform strains to furan aldehydes and efficiently identifying valuable genetic traits are still extremely challenging (Kurgan et al., 2019).

The soil bacterium *Pseudomonas putida* KT2440 (*P. putida* KT2440) is gaining increasing attention. It is a model organism and also a workhorse in biorefinery processes and synthetic biology due to its metabolic versatility, genetic tractability, and inherent tolerance to harsh chemical environments (Li et al., 2019; Henson et al., 2021; Notonier et al., 2021). These advantages make it a particularly valuable chassis for the sustainable biomanufacturing of a wide range of chemicals (Mohamed et al., 2017). Despite this bacterium possesses intrinsic robustness, further improvement of its toxicity tolerance is necessary to achieve effective lignocellulose biorefining processes with pretreated feedstocks that contain a high concentration of inhibitors (Calero et al., 2018). Recently, several studies have attempted to enhance the tolerance of *P. putida* KT2440 to biomass-derived inhibitors. Mohamed et al. obtained a strain with enhanced tolerance toward the aromatic compounds p-coumaric acid and ferulic acid through adaptive laboratory evolution (Mohamed et al., 2020). Ionic liquid tolerant strains were achieved by using a similar approach (Lim et al., 2020). The mechanism behind tolerance toward p-coumaric acid in *P. putida* KT2440 was investigated using transposon insertion sequencing (Calero et al., 2018). However, research focusing on inhibitor tolerance is scarce for *P. putida* KT2440 except for a few reports. Furthermore, as a typical inhibitor in the pretreated feedstock and the vital toxic component to cells of *P. putida* KT2440 (Wang et al., 2017a; Jayakody et al., 2018), further elevated toxicity tolerance and conversion of *P. putida* KT2440 toward furfural will be required to achieve industrial application, whereas little study was focused on the development of furfural-tolerant strain for *P. putida* KT2440. To date, knowledge of its furan aldehydes tolerance mechanism is limited relative to other widely used platform microorganisms, such as *E. coli* and *S. cerevisiae* (Gosset, 2017). Hence, developing a robust furfural-tolerant strain and unveiling its tolerance and metabolic responses to furfural are vitally important for improving our mechanistic understanding to maximize its biotechnological potential.

In this study, the ALE strategy was employed to generate furan aldehydes-resistant *P. putida* strains by serially exposing exponential phase cells to furfural with gradually increasing concentration. Endpoint populations with improved fitness in high concentrations of furfural were subjected to whole-genome resequencing analysis, and the favorable contributions of these mutations identified were validated through reverse engineering. Most of the identified mutations related to ABC transporter and hypothetical protein were found to highly contribute to the improved phenotype. Taken together, the evolved strains and the mutations discovered in this study provided valuable genetic information for future engineering strains to enhance furan aldehydes tolerance and conversion, ultimately improving sustainable lignocellulose-based biomanufacturing.

## Materials and methods

### Reagents and materials

Furfural and HMF purchased from Sigma Chemical. Pretreated hydrolysate of corn stover was obtained from Yigao Biotechnology Co., Ltd (Shanghai, China).

### Bacterial strains and culture conditions

*Pseudomonas putida* KT2440 pK18MS-Δ*gcd*-Δ*gtsABCD* (hereafter *P. putida* ZL), a strain of *P. putida* KT2440 with the destruction of glucose metabolism pathway, was conducted previously in our laboratory as a detoxified bacterium for the detoxification of lignocellulosic hydrolysates (Zou et al., 2021). This strain was further used as the parental strain for adaptive laboratory evolution. For seed propagation and cloning experiments, *P. putida* and *E. coli* strains were routinely pre-cultured overnight in Luria–Bertani (LB) broth.

### Adaptive laboratory evolution experiments

**Figure 1:**
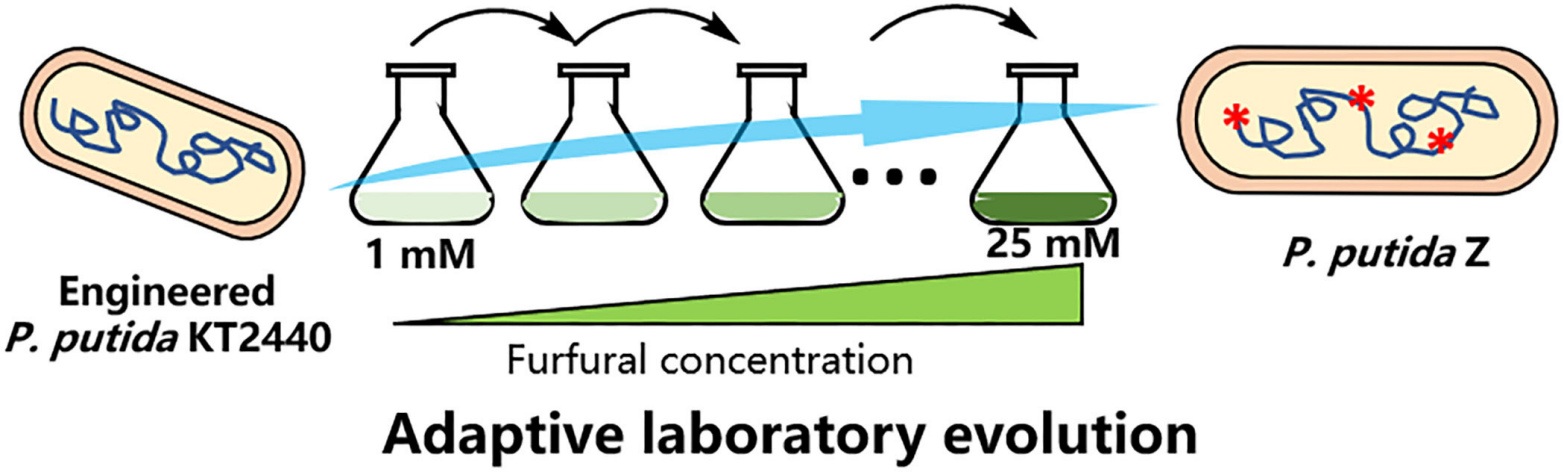
Diagram of adaptive laboratory evolution used in this study.

The procedure of ALE of *P. putida* ZL is shown in Figure 1. The ALE experiment was performed in a 100-ml flask containing 10 ml of 5 g/L of sodium acetate minimal medium supplemented with an increasing concentration of furfural and cultured at 30°C and 200 rpm. First, pre-cultures were grown in the M9 medium containing 5 g/L of sodium acetate. When cell growth was observed (OD_600_ of at least 0.5), the pre-culture at OD_600_ 0.1 was inoculated into a 5 g/L of sodium acetate M9 medium with 1 mM furfural. When stable cell tolerance was reached under the identical concentration of furfural, the cells of OD_600_ 0.1 were passed into a fresh medium with a higher furfural concentration (5 mM). Cells were serially passaged until a significantly enhanced tolerance of furfural (25 mM) was achieved, and then the strains (named *P. putida* Z) were isolated for further analysis.

### Whole-genome sequencing and analysis

The genomic DNA of *P. putida* Z for resequencing was extracted through a Bacteria Genomic DNA Extraction Kit. Raw sequencing reads were obtained on the Illumina sequencing platform by Gene Denovo Biotechnology Co., Ltd (Guangzhou, China). BWA (Li and Durbin, 2009) with default parameters was employed for mapping the reference sequence against the database. SNPs and InDels were analyzed by using *GATK's* UnifiedGenotyper. Detected variants were functionally annotated *via ANNOVAR* (Wang et al., 2010).

### Plasmids and strains construction

Strains, plasmids, and the primers used in this study are shown in Supplementary Tables S1, S2. *Escherichia coli Trans*1-T1 was used for gene cloning. Target genes were amplified from evolved *P. putida* KT2440 chromosomal DNA and wild-type *P. putida* KT2440 genomic DNA, respectively, and subsequently were ligated with the reverse PCR product of the expression vector pBBR1MCS-2. All plasmids were constructed by using the pEASY^®^-Basic Seamless Cloning and Assembly Kit (TransGen Biotech Co., Ltd.). The resulting recombinant plasmids were transformed into chemically competent *E. coli Trans*1-T1 cells according to the manufacturer's instructions. Followed that, the correct recombinant plasmids extracted from *E. coli* were electroporated separately into electrocompetent *P. putida* KT2440 to yield the corresponding expression strains. *Pseudomonas putida* KT2440 harboring the pBBR1MCS-2 empty plasmid was used for the control strain. Agar plates and liquid medium were supplemented with 50 μg/ml of kanamycin for the selection of positive cells.

### Furfural tolerance of recombinant *P. putida* strains

For furfural tolerance experiments of recombinant strains, M9 minimal medium (Dvorák and de Lorenzo, 2018) with a specified concentration of furfural, supplemented with 1% glucose and 50 μg/ml of kanamycin was used for the growth of *P. putida* strains, unless stated otherwise. Overnight cultures of the *P. putida* strains were diluted in the M9 medium to give the initial turbidity of 0.2 under the wavelength of 600 nm. Then the strains were cultivated at 30°C with the agitation of 200 rpm. Samples were taken at regular time intervals for the analysis of cell growth and the consumption of furfural.

### Lignocellulosic hydrolysates experiments

For real hydrolysate experiments, the hydrolysate medium was prepared by adding salt ingredients of the minimal M9 medium (Sambrook et al., 1989) to hydrolysate containing monosaccharides and inhibitors. The hydrolysate medium was adjusted to pH 7.0 with 5 M sodium hydroxide and was autoclaved at 115°C for 10 min before use.

*Pseudomonas putida* strains were pre-cultured in LB, and overnight cultures were harvested by centrifugation for 4 min at 6, 000 *g*, and then resuspended in 25 ml of hydrolysate media in 250 ml Erlenmeyer flasks at an initial OD_600_ of 0.2 and incubated at 30°C with shaking at 200 rpm.

### Cell growth and metabolite analysis

The cell growth was monitored by measuring the turbidity values using a spectrophotometer at 600 nm (OD_600_). The concentration of furfural was analyzed according to the method reported by Yan et al. (2018). The concentrations of glucose, HMF, and sodium acetate were determined as described previously (Zhou et al., 2022).

## Results and discussion

### Adaptive laboratory evolution for improving furan aldehydes tolerance in engineered *P. putida*

**Figure 2:**
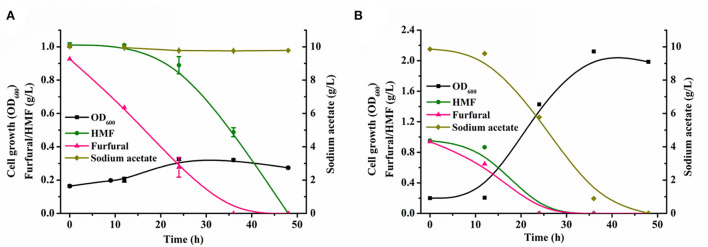
Comparing growth and conversion of inhibitors between original strains and evolved strains in mock lignocellulosic hydrolysate. Growth and inhibitors conversion profiles of the original strains **(A)**, the growth and inhibitors conversion profiles of the evolved strains **(B)** in the minimal medium supplemented with 1 g/L of HMF, 1 g/L of furfural, and 10 g/L of sodium acetate during 48 h cultivation. All data are the mean value ± standard deviation of independent two experiments.

Our previously constructed sugar metabolism blocking strain *P. putida* ZL (Zou et al., 2021) can natively detoxify low concentrations of toxic furan aldehydes in the mock hydrolysate to the corresponding less toxic carboxylic acid, but the conversion rate of furan aldehydes was slow. It required 36 h to completely convert 1 g/L of furfural and 1 g/L of HMF in the presence of 5 g/L of sodium acetate (Supplementary Table S3). When the content of sodium acetate in the mock hydrolysate was elevated to 10 g/L, it took a longer time (48 h) for the strain to achieve full conversion of furan aldehydes (Figure 2A). The presence of furan aldehydes substantially hindered the growth of the strain. Although *P. putida* KT2440 has been extensively studied due to its outstanding tolerance to harmful compounds (Mohamed et al., 2020), this strain is less tolerant to furan aldehyde inhibitors (Calero et al., 2018; Horlamus et al., 2019). In addition, we observed that only when furfural was reduced to a certain level, the concentration of HMF began to decrease significantly (Supplementary Table S3, Figure 2A). This phenomenon indicated that furfural is a more toxic inhibitor for *P. putida* KT2440's metabolism than HMF (Gosset, 2017). The removal of furfural is of major importance before rapid growth and metabolism of carbon source by strain.

To improve the tolerance and conversion of engineered *P. putida* ZL to furan aldehydes, the evolutionary process was executed in the M9 medium comprising 5 g/L of sodium acetate with gradually increasing concentrations of furfural. The strain was first cultivated in a medium with a low concentration of furfural. When the bacterial cell reached the exponential growth phase, it was transferred to the medium containing the equal concentration of furfural at an initial OD_600_ of 0.1, and the transfer was repeated 5–10 times under the same furfural concentration condition until the tolerance was stable. Subsequently, the cells were passed to the fresh medium with a higher furfural concentration. Eventually, the strain can grow stably in the M9 minimal medium containing up to 25 mM furfural after about 4 months of domestication.

The evolved strain with largely improved cell growth and efficient furfural and HMF conversion was observed. The evolved strain transformed 1 g/L of furfural and HMF in 12 h and used 5 g/L of sodium acetate as a carbon source to achieve a maximum growth OD_600_ of 1.32 in 24 h (Supplementary Table S4). A 24 h shortened furan aldehydes conversion and a six-fold improvement in cell growth were observed relative to the parent strain under similar conditions. Additionally, in the growth medium containing 10 g/L of sodium acetate, the evolved strain could fully clear the identical concentration of furan aldehydes within 24 h, and reached a maximum growth OD_600_ of 2.12 using sodium acetate as a sole carbon source (Figure 2B). Even if the furfural concentration in the medium was enhanced to 2 g/L, the strain could still rapidly transform within 24 h and exhibited robust growth (Supplementary Table S5). Even after a three-fold increase in cultivation time, the parental strain did not show any noticeable metabolic activity under the same inhibitor loading conditions (Supplementary Table S6). These evident differences indicated that ALE is a powerful strategy, which enabled the evolved strain to effectively convert and use the components of mock hydrolysate and thereby promoted bacterial growth by alleviating furan aldehydes toxicity under high concentrations of furfural conditions. In addition, these results also showed that relieving cellular furfural toxicity was essential in driving the metabolism of available carbon sources in microbes (Gao et al., 2022).

### Whole-genome resequencing of evolved strains and mutations analysis

Although the evolved strain had improved furfural tolerance and conversion, the associated toxicity tolerance mechanism remains unclear, and the related genes in the cells have not yet been elucidated (Zheng et al., 2020; Henson et al., 2021). To identify mutations and important genes responsible for the acquired furfural tolerance phenotypes and bioconversion in the ALE strain, whole-genome resequencing was performed on the best-evolved strain and then compared with the genome of the wild-type strain (NC_002947.4).

With this approach, we obtained a total of 37 mutations, of which 24 mutations were single-nucleotide polymorphisms (SNPs) and 13 mutations were insertion–deletion polymorphisms (InDels). Among the 24 SNP mutations, four were synonymous, nine were non-synonymous, and one was terminated prematurely (Supplementary Table S7). A total of SNP non-synonymous mutations in eight genes were identified from evolution performed under the ALE conditions (Table 1). Of such mutations, one mutation in gene PP_RS02880 (nucleotide change T517A, amino acid change W173R) encodes an aldehyde dehydrogenase. This gene has been previously reported involved in catalyzing the oxidation reactions of the ethylene glycol to glyoxylate in *P. putida* KT2440 (Li et al., 2019), which is likely related to the oxidation of furan aldehydes. Several other aldehyde dehydrogenases from *Comamonas testosterone* (*C. testosterone*) and *Amorphotheca resinae* ZN1 (*A. resinae* ZN1) have also been found that responsible for the oxidation of furan aldehydes to the less toxic acids (Wang et al., 2015; Zhang et al., 2020). Furthermore, a mutation was found in gene PP_RS13510 (nucleotide change C1283T, amino acid change A428V) that also encodes an aldehyde dehydrogenase. However, the protein sequence encoded by this mutation has only 38.11% identity with the protein sequence encoded by mutated PP_RS02880 using protein blast BLASTP. Three different mutations occurred in gene PP_RS18130 (nucleotide change A67G and T65A, amino acid change R23G as well as I22K) and PP_RS19785 (nucleotide change C20A, amino acid change A7D), respectively. Both of them are annotated as ATP-binding cassette (ABC) transporter substrate-binding proteins. The ABC transporter, as one transporter of the multidrug resistance (MDR) efflux pumps, was found to be crucial for enhancing vanillin tolerance of *S. cerevisiae* (Wang et al., 2017c) and increasing *P. putida*' tolerance toward p-coumaric acid and toluene (Calero et al., 2018), which probably plays a potential tolerance role in the evolved strains in this study. A mutation in gene PP_RS19720 (nucleotide change A988C, amino acid change I330L), encoding a major facilitator superfamily (MFS) transporter. MFS is also a type of MDR efflux pump. Efflux activity is an important mechanism of many bacteria's resistance to toxicants, which can expel a broad range of hydrophobic compounds through the efflux system, thereby sustaining the normal metabolism of cells (Delmar et al., 2014; Jiménez-Bonilla et al., 2020). Several MFS transporters have been overexpressed for improving microbial tolerance toward several toxic compounds, including propionate, toluene, and phenol (Ma et al., 2021). The mutation located in gene PP_RS20740 (nucleotide change G1012T, amino acid change V338F) encodes a hypothetical protein. The function of the gene has not been studied before. The mutation contained in gene PP_RS26785 (nucleotide change G133A, amino acid change E45K) encodes a transcriptional regulator. Transcriptional regulator plays a key role in furfural tolerance, which was found to confer furfural tolerance in mutants of *Z. mobilis* (Tan et al., 2015). The mutation in gene PP_RS02385 (nucleotide change A1046G, amino acid change Y349C), encoding a DNA-directed RNA polymerase subunit beta'. Three different mutations in this locus were previously identified in *E. coli* MG1655 evolved with ionic liquids (Mohamed et al., 2017). Besides, a recently discovered mutation in this locus was reported to improve the tolerance of *E. coli* to furfural (Zheng et al., 2022). However, the role of this gene in *P. putida* remains to be identified. In this study, we speculated that the change in gene PP_RS02385 can alleviate the damage of furfural on bacterial RNA synthesis under furfural stress, thus enhancing the tolerance to furfural.

**Table 1:** Non-synonymous mutations of evolved strain identified by genome resequencing.

**Gene ID**	**Nucleotide alteration**	**Amino acid alteration**	**Gene product**
PP_RS02880	T517A	W173R	Aldehyde dehydrogenase
PP_RS13510	C1283T	A428V	Aldehyde dehydrogenase
PP_RS18130	T65A, A67G	I22K, R23G	ABC transporter substrate-binding protein
PP_RS19720	A988C	I330L	MFS transporter
PP_RS19785	C20A	A7D	Metal ABC transporter substrate-binding protein
PP_RS20740	G1012T	V338F	Hypothetical protein
PP_RS26785	G133A	E45K	Cadmium resistance transcriptional regulator CadR
PP_RS02385	A1046G	Y349C	DNA-directed RNA polymerase subunit beta'

Among the 13 Indel mutations, 11 have no known function type annotation, and the mutations occurred upstream and downstream of structural genes. Another two genes (PP_RS01335 and PP_RS01475) had insertion mutations in exons. The resequencing results showed that the gene PP_RS01335 had an insertion mutation at the 701st position (a base C was inserted). The genome annotation information shows that PP_RS01335 encodes a phosphoenolpyruvate carboxykinase and contains a true frameshift, and thus the open reading frame is classified as a pseudogene. With a single-base insertion, the reading frame of this gene was shifted, which enabled the pseudogene into a gene that encodes a protein of normal function. The enzyme encoded by the gene catalyzes the phosphorylation and decarboxylation of oxaloacetate to form phosphoenolpyruvate using ATP. Although this is an important enzyme in the process of gluconeogenesis, its effect on the conversion of furan aldehydes is unclear. The resequencing results suggested that the gene PP_RS01475 inserted a base T at the 209th position. The genome annotation information shows that the gene PP_RS01475 encodes a protein containing the DUF3077 domain and is classified as a pseudogene (275 bp). Similarly, a single-base insertion mutation was located inside the gene, which enabled the pseudogene into a gene that encodes a normal functional protein.

These mutations detected are likely associated with high tolerance phenotypes and are possibly causal. However, most of such mutations have not been reported in previous studies. All of these provide some useful information for inverse metabolic engineering. Detailed investigations to uncover their mechanism of causality are warranted.

### The effect of overexpression of key targets on *P. putida* tolerance to furfural

To determine if the above-mentioned mutations are responsible for improved furfural tolerance, the eight genes with SNP mutations were separately overexpressed *via* plasmid in the wild-type strain *P. putida* KT2440. Subsequently, the growth and the furfural transformation profiles of the constructed recombinant strains were characterized in the M9 minimal medium containing around 20 mM furfural.

**Figure 3:**
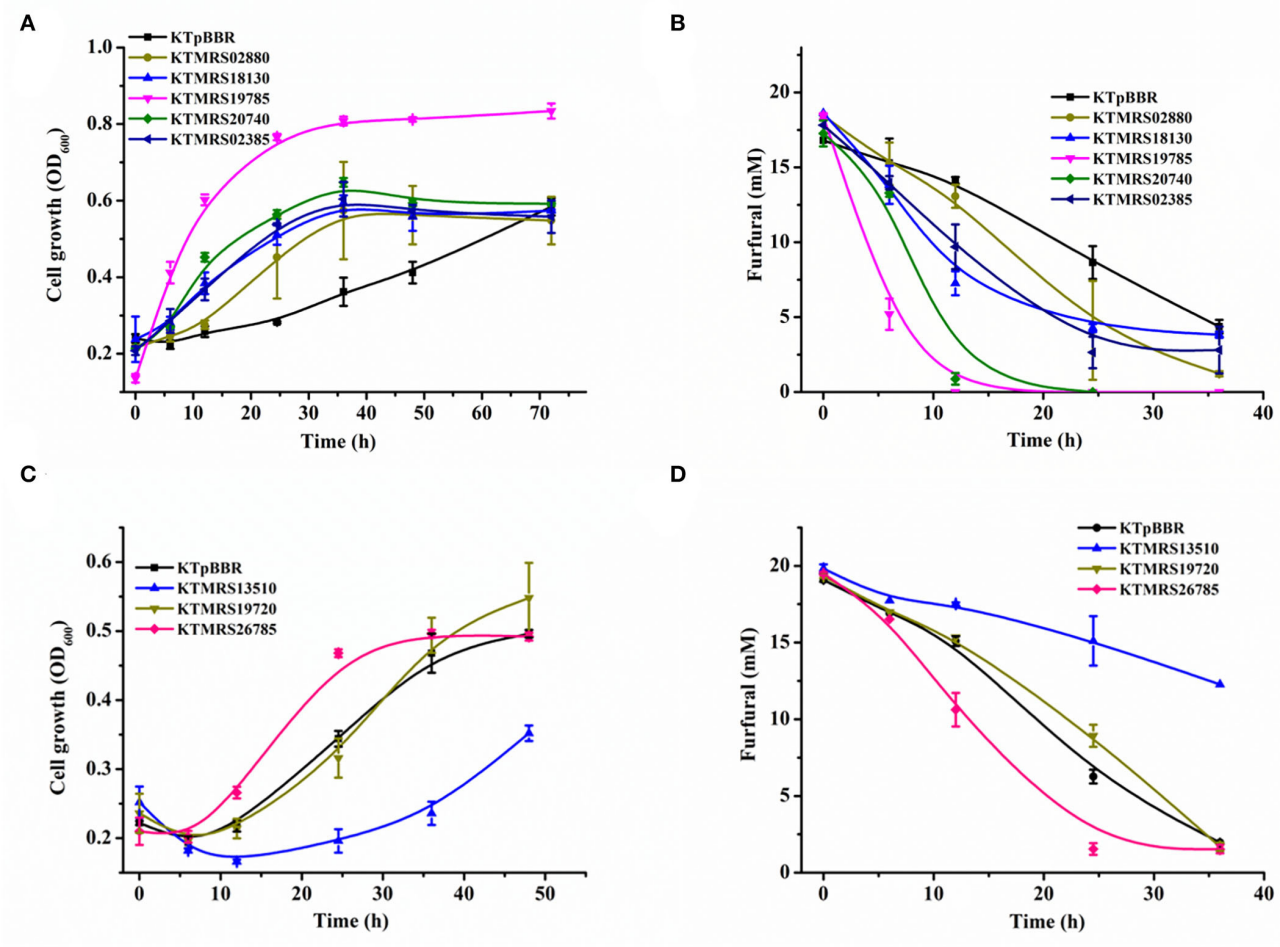
Effects of mutations on growth and furfural tolerance of strains. The growth curve **(A)** and furfural conversion course **(B)** of strains overexpressing MRS02880, MRS18130, MRS19785, MRS20740, MRS02385, and the control (pBBR1MCS2); The growth curve **(C)** and furfural conversion course **(D)** of strains overexpressing MRS13510, MRS19720, MRS26785, and the control (pBBR1MCS2). All data are the mean value ± standard deviation of independent two experiments.

As shown in Figure 3, the overexpression of PP_MRS19785, PP_MRS20740, PP_MRS18130, PP_MRS02385, PP_MRS02880 as well as PP_MRS26785 improved cell growth and enhanced the furfural conversion over the control strain, in which the overexpression of PP_MRS19785 showed the most significant enhancement. About 18 mM of furfural was completely converted in 12 h (Figure 3B), along with remarkable cell growth. An OD_600_ of 0.816 was reached in 36 h (Figure 3A). The equal concentration of furfural was fully transformed within 24 h after PP_MRS20740 was overexpressed, leading to substantial cell growth, and a maximum growth OD_600_ of 0.65 was achieved in 36 h (Figure 3). Moreover, several other mutant strains, including KTMRS18130, KTMRS02880, KTMRS02385, and KTMRS26785, also showed significant growth and inhibitor conversion advantages compared to the control under equal furfural stress, which was 1.81-, 1.60-, 1.91-, and 1.36-fold that of the control in biomass concentration, respectively, in 24 h, despite the performance was slightly worse than the mutant strains KTMRS19785 and KTMRS20740. These results suggested that the overexpression of such mutated genes was crucial for the growth and conversion of the strains when a high concentration of furfural was present. However, the recombinant strain KTMRS19720 did not show improvement in the cell growth and the conversion of furfural during the whole culture process relative to the control strain (Figures 3C,D). Despite PP_RS19720 being annotated as an MFS transporter, we did not observe a noticeable improvement in its tolerance to furfural, which may be a result of the substrate specificity of efflux pumps (Jayakody et al., 2022). Overexpression of PP_MRS13510 resulted in the strain KTMRS13510 being inferior to the control strain in the cell growth and furfural conversion (Figures 3C,D), which might be attributed to the extra metabolic stress from the overexpression of unwanted proteins (Jiménez-Bonilla et al., 2020). Based on these results, the strains KTMRS19720 and KTMRS13510 were excluded to simplify the genetic analysis, and the rest of the mutant strains were selected for further study.

**Figure 4:**
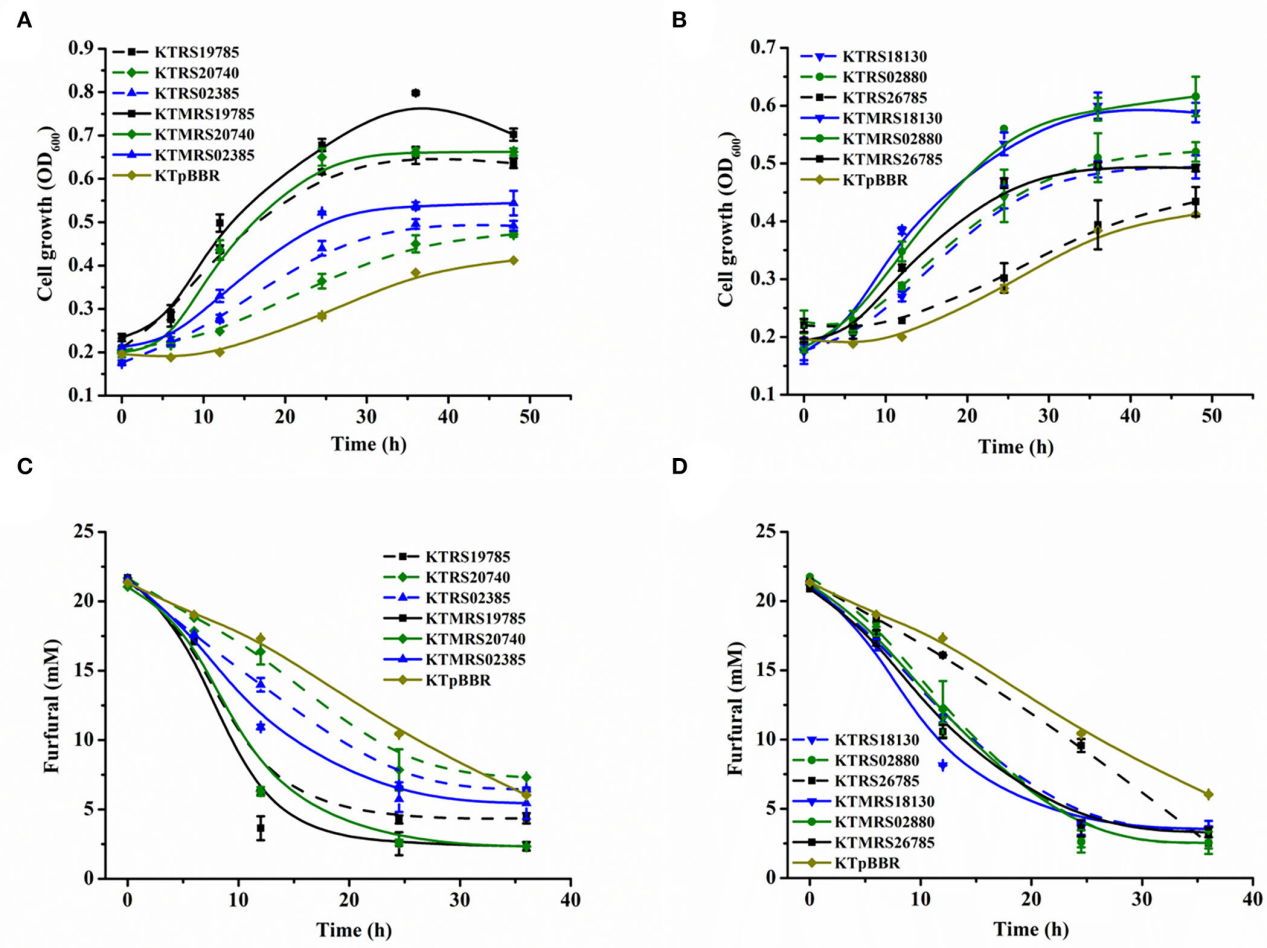
Comparison of the effects of strains overexpressing wild-type genes and overexpressing mutated genes on furfural tolerance. The growth curve **(A)** and furfural conversion **(C)** of strains overexpressing wild-type genes and overexpressing mutated genes PP_RS19785, PP_RS20740, PP_RS02385, and the control (pBBR1MCS2); The growth curve **(B)** and furfural conversion **(D)** of strains overexpressing wild-type genes and overexpressing mutated genes PP_RS18130, PP_RS02880, PP_RS26785, and the control (pBBR1MCS2). All data are the mean value ± standard deviation of independent two experiments.

To further investigate if the improved furfural tolerance phenotype was actually due to the presence of mutation sites, the wild-type gene and the mutation site-containing gene were overexpressed separately in *P. putida* KT2440, and then their tolerance to furfural was also evaluated and compared. As shown in Figure 4, all the tested strains overexpressing the wild or mutated genes outperformed the control strain in terms of cell growth and transformation of the inhibitor furfural. This result suggested that the overexpression of both wild-type and mutated genes was beneficial for the tolerance of strain to furfural. Moreover, we observed that the presence of the mutation site could further improve the growth and furfural transformation of the overexpressed strain under furfural pressure. These results illustrated that the mutation in the gene plays a positive role in the tolerance and conversion of the strain toward furfural. It is noteworthy that the strain overexpressing the PP_RS19785 wild-type gene also showed great growth and excellent furfural conversion performance, which can convert 80% of the starting furfural and reach a maximum growth OD_600_ of 0.654 in 36 h (Figures 4A,C). The transformation performance was comparable to that of the KTMRS20740 strain overexpressing the mutated gene. This result indicates that the gene PP_RS19785 is intrinsically highly tolerant to toxic furfural. To the best of our knowledge, there is no prior research inspecting the roles of these genes and mutations in the furfural tolerance of this bacterium, which makes these findings a novel discovery.

The above results suggested that the genes responsible for furfural tolerance were diverse in *P. putida* KT2440 (Bilal Jilani et al., 2021). The transport process related to the function of the ABC transporter appears to be the main furfural tolerance mechanism in this strain. A previous study shows that the expression of small multidrug resistance (SMR) pumps, SugE and MdtJI, increases *E. coli* tolerance to furfural by expelling furfural from cells (Kurgan et al., 2019). But in this study, we did not detect the increased extracellular furfural concentrations over time when the strains overexpressing ABC transporter were incubated with furfural, suggesting that the increased tolerance of this strain was probably not due to the efflux of furfural (Bitzenhofer et al., 2021). Moreover, the ABC transporter system has been proposed to be engaged in the maintenance of outer membrane stability in the presence of membrane disruptors (Calero et al., 2018). A different ABC transporter Ttg2ABC associated with the membrane process in *P. putida* KT2440 has been described that enhancing p-coumaric acid tolerance (Calero et al., 2018). Previous studies have shown that furfural disrupts cell membranes (Allen et al., 2010; Zheng et al., 2022), leading to the loss of essential membrane functions (Bitzenhofer et al., 2021); we, therefore, reasoned that the cell envelope stress response mechanism is also essential for furfural tolerance of this strain. Furthermore, the uncharacterized protein, aldehyde dehydrogenase, transcriptional regulator, and RNA polymerase subunit beta' were also effective in conferring furfural tolerance of this bacterium.

**Figure 5:**
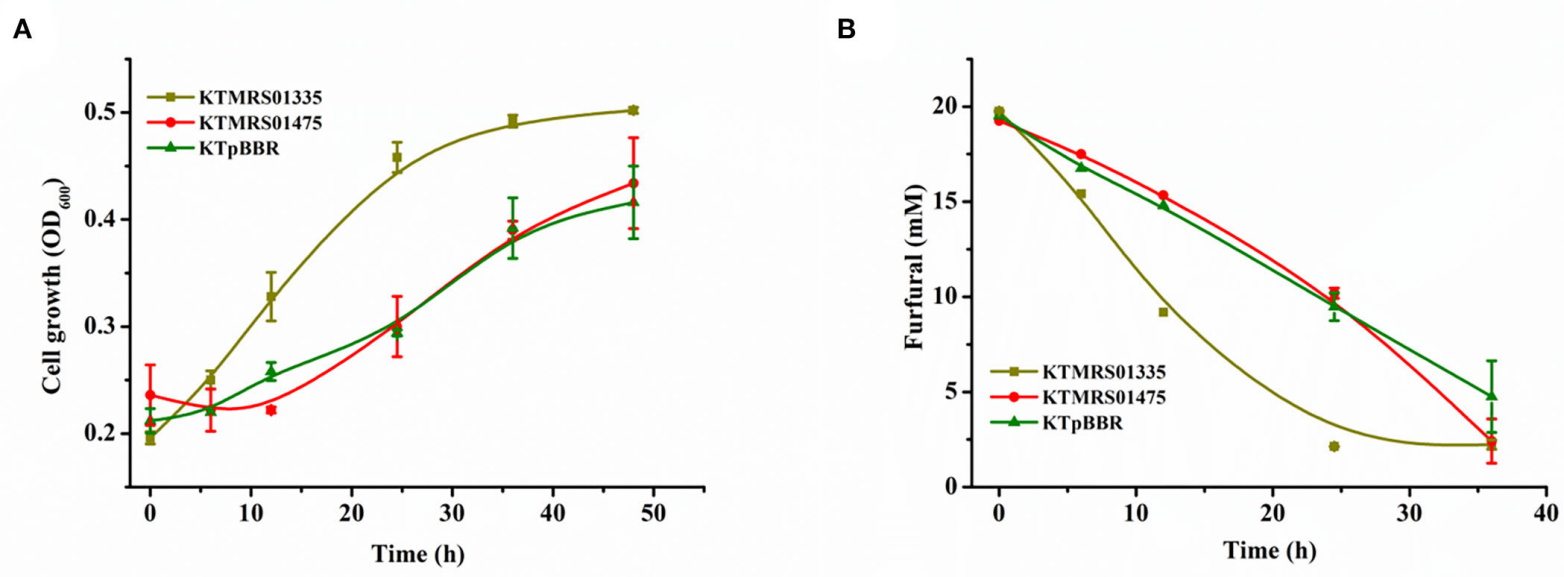
Effects of strains overexpressing Indel genes on furfural tolerance. The cell growth curve **(A)** and furfural conversion **(B)**. All data are the mean value ± standard deviation of independent two experiments.

Separately, two genes screened from Indel mutations were also further characterized. Strains overexpressing these two mutations were also tested in the M9 minimal medium containing ~20 mM furfural. Compared with the control, the strain KTMRS01335 overexpressing the mutated gene PP_MRS01335 showed obvious growth and the conversion of furfural (Figure 5). The strain KTMRS01335 can achieve the transformation of 10.6 mM furfural within 12 h, whereas the control strain can only achieve 4.7 mM, which was 2.2 times that of the control strain. However, the expression of PP_MRS01475 did not exert a positive effect.

Based on the growth and furfural conversion analysis, we next characterized the furan aldehydes conversion performance of strains KTMRS19785, KTMRS20740, KTMRS18130, KTMRS02880, KTMRS02385, KTMRS26785, and KTMRS01335 in real hydrolysate.

### Overexpression of ABC transporters and hypothetical protein leads to noticeably improved *P. putida* tolerance to real pretreated biomass hydrolysate

**Figure 6:**
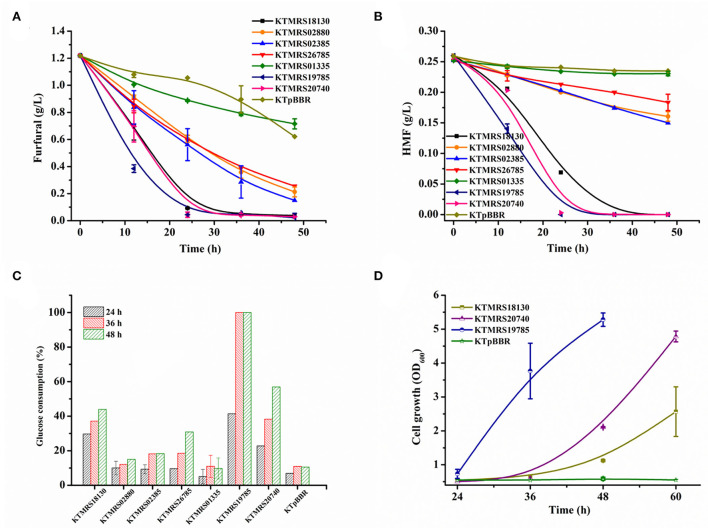
Performance evaluation of recombinant strains in real hydrolysate. The conversion of furfural **(A)** and HMF **(B)** of recombinant strains; glucose consumption profiles **(C)** of recombinants and the growth of representative recombinants **(D)** during the period of furfural and HMF conversion; the verification was conducted in corn stover hydrolysate medium. All data are the mean value ± standard deviation of independent duplicate.

Compared with a single furfural, lignocellulosic hydrolysate containing multiple inhibitors has stronger toxicity to microbial metabolism (Ouyang et al., 2020; Millán Acosta et al., 2021). The toxicity of hydrolysate is a major hurdle to its utilization. We thus further examined the tolerance performance of recombinants in the real hydrolysate. The hydrolysate used in this study comprises glucose (around 10 g/L), xylose (16.94 g/L), arabinose (2.29 g/L), acetic acid (3.04 g/L), formic acid (0.99 g/L), levulinic acid (0.68 g/L), furfural (1.22 g/L), and HMF (0.26 g/L) as well as vanillin, 4-hydroxybenzaldehyde, syringic acid, and other inhibitors. As shown in Figures 6A,B, compared with the control strain, all test strains overexpressing mutated genes exhibited better transformation ability of furan aldehydes. The strain KTMRS19785 had the highest conversion capacity, with 68% of the initial furfural and 46% of the starting HMF reduced at 12 h, and nearly complete conversion of furfural and HMF at 24 h. However, the control strain only achieved 17.4% furfural and about 6.9% HMF conversion after 24 h of cultivation. The strain KTMRS20740 could also completely convert furfural and HMF in about 24 h, despite the transformation of furan aldehydes in the early stage of the strain being slightly slower than that of the strain MRS19785. The gene PP_RS20740 encodes a hypothetical protein of unknown function, which deserves further investigation given its great potential in enhancing the tolerance of strains to furan aldehydes. Strain KTMRS18130 presented a similar furfural conversion trend as strain KTMRS20740, whereas it is slightly slower than KTMRS20740 for the conversion of HMF. In addition, the strains KTMRS02385, KTMRS02880, and KTMRS26785 also displayed excellent furfural conversion capacity, as they can reduce furfural in hydrolysate to a very low level within 48 h (Figure 6A). A significant reduction in HMF by these strains was observed compared to the control strain, but the transformation rate was slightly inferior to the three bacteria mentioned above (Figure 6B).

Meanwhile, the glucose consumption and cell growth were evaluated during furan aldehydes conversion. Figure 6C shows the glucose consumption by recombinant *P. putida* strains. The strain KTMRS19785 showed the highest glucose consumption, with ~41% of the glucose consumed at 24 h, 100% of the glucose used up, and a growth OD_600_ of 3.76 (Figure 6D) achieved in 36 h, representing a nearly six-fold increase in biomass concentration compared to the parental strain. The strain KTMRS18130 consumed 30% of the glucose at 24 h. 44% of the glucose consumption and OD_600_ of 1.1 (Figure 6D) of the cell growth were detected at 48 h. The strain KTMRS20740 depleted 23% of the glucose at 24 h and 57% of the glucose consumption and cell growth of OD_600_ 2.1 (Figure 6D) were measured at 48 h. A markedly faster glucose consumption rate was observed than the strain KTMRS18130 in the last 12 h. The remaining strains (except for KTMRS01335) also had significant consumption of glucose than the control strain during this process but the consumption was not as obvious as the first three strains. The metabolic results of these strains for glucose are consistent with their transformation performance for furan aldehydes inhibitor.

The transformation performance of these recombinant strains in real hydrolysate contains a mixture of inhibitory compounds, indicating that mutations that increase furfural tolerance are also useful for increasing bacterial tolerance to pretreatment hydrolysate. The overexpression of mutated genes related to ABC transporters (PP_RS19785 and PP_RS18130) and a hypothetical protein (PP_RS20740) significantly promoted the furan aldehydes conversion, reducing the overall toxicity of the hydrolysate at varying degrees and improving bacterial growth. Taken together, such findings will greatly reinforce our capability to engineer robust microbial cell factories for enhanced tolerance of lignocellulose-derived microbial inhibitors, thereby improving the bioconversion of lignocellulosic hydrolysates to valuable products (Bitzenhofer et al., 2021).

## Conclusion

In this study, we obtained a highly furfural-tolerant strain through adaptive evolution. Whole-genome resequencing analysis identified 10 candidate genes associated with 11 mutations in the evolved strain that were potentially responsible for the enhancement of furan aldehyde tolerance. We confirmed that most of these genes and mutations are beneficial for furfural tolerance and conversion *via* reverse engineering. In particular, three of the mutated genes, including PP_RS19785 and PP_RS18130 (both of them encoding ABC transporters) as well as PP_RS20740 (encoding a hypothetical protein), exhibited the best furfural conversion performance, which is the key contributor to improved furfural tolerance in ALE strains. Furthermore, strains overexpressing these three mutations substantially elevated the fermentability of toxic hydrolysate. Overall, the crucial mutations identified and the genomic insights into the potential tolerance mechanism of furfural in the present study would be beneficial for resolving the lignocellulosic fermentation inhibitor problem and contribute to engineering industrial microorganisms to produce various chemicals and fuels from toxic lignocellulosic hydrolysates.

## Data availability statement

The original contributions presented in the study are publicly available. This data can be found here: NCBI, PRJNA855347.

## Author contributions

LZ: conceptualization, investigation, data curation, formal analysis, visualization, and writing—original draft. XJ and YT: methodology and visualization. ZZ: conceptualization and writing—review and editing. JO: conceptualization, writing—review and editing, funding acquisition, and supervision. All authors contributed to the article and approved the submitted version.

## Funding

This study was supported by the National Key Research and Development Program of China (2018YFA0902200) and the National Natural Science Foundation of China (22078163).

## Conflict of interest

The authors declare that the research was conducted in the absence of any commercial or financial relationships that could be construed as a potential conflict of interest.

## Publisher's note

All claims expressed in this article are solely those of the authors and do not necessarily represent those of their affiliated organizations, or those of the publisher, the editors and the reviewers. Any product that may be evaluated in this article, or claim that may be made by its manufacturer, is not guaranteed or endorsed by the publisher.
