# Supplementary material for: Unraveling the mechanism of furfural tolerance in engineered Pseudomonas putida by genomics
Source: Front Microbiol. 2022 Oct 20;13:1035263. doi: 10.3389/fmicb.2022.1035263 (PMC9630843; doi:10.3389/fmicb.2022.1035263)
Supplement: Supplementary file 1 [file Data_Sheet_1.docx]

Supplementary files for

**Unraveling the mechanism of furfural tolerance in engineered *Pseudomonas putida* by genomics**

Lihua Zou, Xinzhu Jin, Yuanming Tao, Zhaojuan Zheng, Jia Ouyang^*^

Jiangsu Co-Innovation Center of Efficient Processing and Utilization of Forest Resources, College of Chemical Engineering, Nanjing Forestry University, Nanjing 210037, People’s Republic of China

^*^Corresponding author. Address: College of Chemical Engineering, Nanjing Forestry University, Nanjing 210037, People’s Republic of China, Tel.: 86-025-85427129, Fax: 86-025-85427587, E-mail: [hgouyj@njfu.edu.cn](mailto:hgouyj@njfu.edu.cn).

Table S1 Plasmids and strains employed in this study.

| Strain or plasmid | Description | Source |
| --- | --- | --- |
| Strains |  |  |
| *Escherichia coli* | Cloning host | TransGen Biotech |
| *Pseudomonas putida* |  |  |
| KT2440 | Wild-type strain, spontaneous restriction-deficient derivative of strain mt-2 cured of the TOL plasmid pWW0 | Lab stock |
| KTpBBR | KT2440 harboring plasmid pBBR1MCS2 | This study |
| KTMRS18130 | KT2440 harboring plasmid pBBR1MCS2-MRS18130 | This study |
| KTMRS02880 | KT2440 harboring plasmid pBBR1MCS2-MRS02880 | This study |
| KTMRS02385 | KT2440 harboring plasmid pBBR1MCS2-MRS02385 | This study |
| KTMRS20740 | KT2440 harboring plasmid pBBR1MCS2-MRS20740 | This study |
| KTMRS26785 | KT2440 harboring plasmid pBBR1MCS2-MRS26785 | This study |
| KTMRS19785 | KT2440 harboring plasmid pBBR1MCS2-MRS19785 | This study |
| KTMRS01335 | KT2440 harboring plasmid pBBR1MCS2-MRS01335 | This study |
| KTMRS01475 | KT2440 harboring plasmid pBBR1MCS2-MRS01475 | This study |
| KTMRS13510 | KT2440 harboring plasmid pBBR1MCS2-MRS13510 | This study |
| KTMRS19720 | KT2440 harboring plasmid pBBR1MCS2-MRS19720 | This study |
| KTRS18130 | KT2440 harboring plasmid pBBR1MCS2-RS18130 | This study |
| KTRS02880 | KT2440 harboring plasmid pBBR1MCS2-RS02880 | This study |
| KTRS19785 | KT2440 harboring plasmid pBBR1MCS2-RS19785 | This study |
| KTRS20740 | KT2440 harboring plasmid pBBR1MCS2-RS20740 | This study |
| KTRS02385 | KT2440 harboring plasmid pBBR1MCS2-RS02385 | This study |
| KTRS26785 | KT2440 harboring plasmid pBBR1MCS2-RS26785 | This study |
| Plasmids |  |  |
| pBBR1MCS2 | A shuttle expression vector, Km^R^ | Lab stock |
| pBBR1MCS2-MRS18130 | pBBR1MCS2 with PP_RS18130 gene from evolved KT2440 | This study |
| pBBR1MCS2-MRS02880 | pBBR1MCS2 with PP_RS02880 gene from evolved KT2440 | This study |
| pBBR1MCS2-MRS02385 | pBBR1MCS2 with PP_RS02385 gene from evolved KT2440 | This study |
| pBBR1MCS2-MRS20740 | pBBR1MCS2 with PP_RS20740 gene from evolved KT2440 | This study |
| pBBR1MCS2-MRS26785 | pBBR1MCS2 with PP_RS26785 gene from evolved KT2440 | This study |
| pBBR1MCS2-MRS19785 | pBBR1MCS2 with PP_RS19785 gene from evolved KT2440 | This study |
| pBBR1MCS2-MRS01335 | pBBR1MCS2 with PP_RS01335 gene from evolved KT2440 | This study |
| pBBR1MCS2-MRS01475 | pBBR1MCS2 with PP_RS01475 gene from evolved KT2440 | This study |
| pBBR1MCS2-MRS13510 | pBBR1MCS2 with PP_RS13510 gene from evolved KT2440 | This study |
| pBBR1MCS2-MRS19720 | pBBR1MCS2 with PP_RS19720 gene from evolved KT2440 | This study |
| pBBR1MCS2-RS18130 | pBBR1MCS2 with PP_RS18130 gene from wild-type KT2440 | This study |
| pBBR1MCS2-RS02880 | pBBR1MCS2 with PP_RS02880 gene from wild-type KT2440 | This study |
| pBBR1MCS2-RS19785 | pBBR1MCS2 with PP_RS19785 gene from wild-type KT2440 | This study |
| pBBR1MCS2-RS20740 | pBBR1MCS2 with PP_RS20740 gene from wild-type KT2440 | This study |
| pBBR1MCS2-RS02385 | pBBR1MCS2 with PP_RS02385 gene from wild-type KT2440 | This study |
| pBBR1MCS2-RS26785 | pBBR1MCS2 with PP_RS26785 gene from wild-type KT2440 | This study |

Table S2 Oligonucleotides primers used in this study.

| Primer | Sequence (5’-3’) |
| --- | --- |
| pBB.f | AGCTGTTTCCTGTGTGAAATTGTTA |
| pBB.r | GCGTTAATATTTTGTTAAAATTC |
| PP_RS13510.f | ATTTCACACAGGAAACAGCTATGTACACTCTCGAATTCTG |
| PP_RS13510.r | TTTTAACAAAATATTAACGCTCAGCCGCGCAACTGGTACC |
| PP_RS19720.f | ATTTCACACAGGAAACAGCTATGTCCATTCTTTCGTGGTC |
| PP_RS19720.r | TTTTAACAAAATATTAACGCTTAAAGATCTCCCCGCGCAA |
| PP_RS02880.f | ATTTCACACAGGAAACAGCTATGCGTTATGCACATCCCGG |
| PP_RS02880.r | TTTTAACAAAATATTAACGCCTAGAAGAAGCCCAGCGGAT |
| PP_RS18130.f | ATTTCACACAGGAAACAGCTATGGGCCGCCTGGGGAATTG |
| PP_RS18130.r | TTTTAACAAAATATTAACGCTCAAGGGCTGGCCGGCACTG |
| PP_RS19785.f | ATTTCACACAGGAAACAGCTATGTTTCGCTCCGCCCTCGC |
| PP_RS19785.f’ | ATTTCACACAGGAAACAGCTATGTTTCGCTCCGCCCTCGA |
| PP_RS19785.r | TTTTAACAAAATATTAACGCTCATGCGCGGTTCTCCTGAA |
| PP_RS20740.f | ATTTCACACAGGAAACAGCTGTGAACGATATGGACGTCAA |
| PP_RS20740.r | TTTTAACAAAATATTAACGCCTATTGAGCTATACGCATTG |
| PP_RS26785.f | ATTTCACACAGGAAACAGCTATGAAGATCGGAGAACTGGC |
| PP_RS26785.r | TTTTAACAAAATATTAACGCTCAATGCCCGTGACTCCGCC |
| PP_RS02385.f | ATTTCACACAGGAAACAGCTTTGAAAGACCTACTGAATTTGCTGA |
| PP_RS02385.r | TTTTAACAAAATATTAACGCTTAATTACCGCTGGAATTCAGCGCT |
| PP_RS01335.f | ATTTCACACAGGAAACAGCTATGACGCAAGCCAACAACAC |
| PP_RS01335.r | TTTTAACAAAATATTAACGCTTACAGCTGCGGGCCAGCGG |
| PP_RS01475.f | ATTTCACACAGGAAACAGCTATGAAAAAAGATGTTTCTGA |
| PP_RS01475.r | TTTTAACAAAATATTAACGCCTAATGCGCCTTACCCTTGG |

RS19785.f' is the upstream primer for amplifying the PP_RS19785 mutated gene sequence while RS19785.f is the upstream primer for amplifying the PP_RS19785 wild-type gene sequence.

Table S3 The growth and inhibitors conversion by wild-type engineered *P. putida* in M9 minimal medium containing 1 g/L furfural, 1 g/L HMF and 5 g/L sodium acetate.

| Time  (h) | OD_600_ | Furfural  (g/L) | HMF  (g/L) | Sodium acetate  (g/L) |
| --- | --- | --- | --- | --- |
| 0 | 0.18±0.00 | 0.89±0 | 0.96±0.01 | 5.01±0.01 |
| 24 | 0.22±0.00 | 0.26±0.03 | 0.90±0.20 | 4.67±0.10 |
| 36 | 0.27±0.00 | 0±0 | 0±0 | 4.38±0.06 |

Table S4 The growth and inhibitors conversion by evolved engineered *P. putida* in M9 minimal medium containing 1 g/L furfural, 1 g/L HMF and 5 g/L sodium acetate.

| Time  (h) | OD_600_ | Furfural  (g/L) | HMF  (g/L) | Sodium acetate  (g/L) |
| --- | --- | --- | --- | --- |
| 0 | 0.2±0.00 | 0.94±0 | 0.95±0.01 | 5.20±0.01 |
| 12 | 0.25±0.00 | 0±0 | 0±0 | 4.04±0.01 |
| 24 | 1.32±0.00 | 0±0 | 0±0 | 0±0 |

Table S5 The growth and inhibitors conversion by evolved engineered *P. putida* in M9 minimal medium containing 2 g/L furfural, 1 g/L HMF and 5 g/L sodium acetate.

| Time  (h) | OD_600_ | Furfural  (g/L) | HMF  (g/L) | Sodium acetate  (g/L) |
| --- | --- | --- | --- | --- |
| 0 | 0.2±0.00 | 2.11±0.12 | 1.10±0.00 | 5.27±0 |
| 24 | 1.06±0.07 | 0±0.00 | 0±0.00 | 2.34±0.11 |

Table S6 The growth and inhibitors conversion course by wild-type engineered *P. putida* in M9 minimal medium containing 2 g/L furfural, 1 g/L HMF and 5 g/L sodium acetate.

| Time  (h) | OD_600_ | Furfural  (g/L) | HMF  (g/L) | Sodium acetate  (g/L) |
| --- | --- | --- | --- | --- |
| 0 | 0.16±0.01 | 1.99±0.01 | 1.03±0.01 | 4.82 |
| 24 | 0.12±0.01 | 1.79±0.02 | 0.99±0.01 | 4.67 |
| 48 | 0.18±0.01 | 1.62±0.04 | 0.98±0.01 | 4.74 |
| 72 | 0.15±0.00 | 1.54±0.06 | 0.99±0.01 | 4.87 |

Table S7 List of all mutations found in sequenced strains. Numbers of found Single Nucleotide Polymorphisms (SNP) and Insertion-Deletion polymorphisms (InDel) and their functional class, divided by the cause of mutation, are shown.

| Sum of all mutations | SNP | | | | InDel | |
| --- | --- | --- | --- | --- | --- | --- |
|  | intergenic | nonsynonymous | stopgain | synonymous | intergenic | unknown |
| 37 | 10 | 9 | 1 | 4 | 11 | 2 |
